# Disentangling the effects of cannabis and cigarette smoking on impulsivity

**DOI:** 10.1177/0269881120926674

**Published:** 2020-06-10

**Authors:** Jason T Round, Therese E Fozard, Amanda A Harrison, Katerina Z Kolokotroni

**Affiliations:** 1 Leeds School of Social Sciences, Leeds Beckett University, Leeds, UK; 2 Faculty of Medicine & Health, University of Leeds, Leeds, UK

**Keywords:** Trait impulsivity, behavioural impulsivity, cannabis, δ-9-tetrahydrocannibinol (THC), cannabinoid receptor partial agonist, tobacco, nicotine, nicotinic acetylcholine receptor agonist, drug abuse, humans

## Abstract

**Background:**

Cannabis smoking and cigarette smoking often co-occur, yet limited research has investigated the potentially different role impulsivity may play when these behaviours occur in isolation, compared with in combination.

**Aims:**

This study examined trait and behavioural impulsivity as a function of both cigarette and cannabis smoking.

**Methods:**

Trait impulsivity (BIS-11) was compared between 44 non-smokers, 76 cigarette only, 47 cannabis only and 58 cannabis plus cigarette smokers. The effects of cigarette and cannabis smoking on behavioural impulsivity (stop-signal and information sampling tasks) were then assessed in 87 of these participants during a laboratory session.

**Results:**

Trait impulsivity was significantly higher in cigarette smokers than non-smokers, irrespective of cannabis use, except for motor impulsivity, where cigarette smoking was only associated with elevated trait impulsivity in non-smokers of cannabis. Dimensions of trait impulsivity were significantly positively related to cigarette smoking frequency and nicotine dependence, but not to cannabis smoking frequency or dependence. Smoking cigarettes or cannabis was associated with significantly impaired reflection impulsivity relative to not smoking either substance. However, no additional increases in reflection impulsivity were observed in those who smoked both cigarettes and cannabis. No group differences in response inhibition were detected.

**Conclusions:**

Heightened trait impulsivity appears to be uniquely related to cigarette smoking, whilst the smoking of cigarettes or cannabis is associated with impairments in reflection impulsivity. Improved outcomes for treating cannabis dependence may result from encouraging concomitant cigarette smokers to cease using both drugs simultaneously in order to reduce heightened impulsivity and risk of relapse.

## Introduction

Cannabis is the most widely used illicit substance globally (UNODC, 2018). In England, primary cannabis use accounts for the largest proportion of young people in treatment for substance misuse (Niblett, 2018). A major factor contributing to cannabis dependence may be co-dependence on nicotine, since many cannabis smokers also smoke cigarettes (Hindocha et al., 2016; Rabin and George, 2015), and are more likely to relapse to cannabis whilst continuing to smoke cigarettes (Haney et al., 2013). Furthermore, co-morbid cannabis-cigarette smoking is associated with heavier cigarette smoking relative to cigarette smoking only (Agrawal et al., 2011), while cigarette smoking mediates the relationship between cannabis use and cannabis dependence (Hindocha et al., 2015). Failure to consider the role of cigarette smoking when attempting to understand factors underlying problematic cannabis use may have hindered research progress. To date, limited research has attempted to disentangle the effects of cannabis smoking from cigarette smoking; in particular, the role impulsivity has in supporting dependence on these substances. Distinguishing between cannabis only smokers and those who smoke both cannabis and cigarettes may be a critical step towards this goal.

Impulsivity is a multifaceted construct encapsulating a range of trait and behavioural characteristics, where delayed rewards are discounted in favour of immediate gratification, actions are prematurely conceived without adequate forethought, and decision-making often lacks reflection despite resulting in negative consequences (Robbins et al., 2012). Moreover, impulsivity is thought to be involved in the transition to addiction for several drugs including cannabis (for reviews see de Wit, 2009; Wrege et al., 2014). Whilst heightened impulsiveness may predispose individuals to initiating substance use (e.g. Vergés et al., 2019), prolonged drug use may further reduce impulse control through drug-induced neuroadaptations, whereby dopaminergic prefrontal cortical processing becomes increasingly subverted by greater subcortical automatic conditioned responding, resulting in persistent drug-taking and making drug-related stimuli highly salient to users (Berridge and Robinson, 2016; Everitt et al., 2008; Goldstein and Volkow, 2002; Piazza and Deroche-Gamonet, 2013).

Trait impulsivity on the Barratt Impulsiveness Scale (BIS) is elevated in several dependent drug user groups relative to controls (e.g. Bozkurt et al., 2013; Ersche et al., 2008). Research has been less consistent in relation to whether trait impulsivity is elevated in cannabis smokers compared with non-smokers (NS). Some studies support this view (Dervaux et al., 2010; Moreno et al., 2012), while others indicate no trait differences between dependent (Johnson et al., 2010) or non-dependent cannabis smokers and NS (Clark et al., 2009; Field et al., 2006). A possible factor accounting for these discrepant findings is that studies have not always controlled for tobacco cigarette smoking in cannabis smokers sampled. In consideration of cigarette smoking, Beaton et al. (2014) found cigarette only smokers and cannabis smokers who smoke cigarettes both display elevated trait impulsivity on the BIS relative to NS and cannabis only smokers – who did not differ from each other – suggesting cigarette rather than cannabis smoking was associated with heightened impulsivity. In contrast, Gilman et al. (2015) found elevated trait motor and non-planning impulsivity in cannabis only smokers relative to NS. In terms of potential additive effects of cannabis and cigarette smoking on impulsivity, none were suggested in the work of Beaton et al. (2014), but these could not be assessed by Gilman et al. (2015) as no cigarette smokers were included in this study. Furthermore, behavioural impulsivity was not measured in either study.

Behavioural impulsivity is principally conceptualised as manifesting in two forms: response inhibition (the ability to inhibit a pre-potent response) and impulsive choice (a preference for smaller immediate over larger delayed reward), which have been the most widely studied aspects of impulsive behaviour to date (Weafer and de Wit, 2013). While evidence indicates that response inhibition, measured by the stop-signal task (SST), is impaired following acute cannabis administration (Ramaekers et al., 2006), it is unknown whether impulsive behaviour is elevated in cannabis smokers residually following long-term use. When not acutely intoxicated, existing evidence suggests that cannabis smokers do not differ from NS on response inhibition on the SST (Filbey and Yezhuvath, 2013; Grant et al., 2012), however neither of these studies controlled for cigarette smoking in the interpretation of task findings. Evidence also indicates that those who smoke both cannabis and cigarettes do not differ from NS on impulsive choice, measured using a delay discounting task (Johnson et al., 2010). Interestingly, in this study cigarettes smoked per day but not bouts of cannabis smoked per day predicted greater discounting of reward, suggesting impulsive behaviour more closely relates to cigarette smoking. However, no study has extended beyond this when comparing the role of cigarette smoking and cannabis use in impulsive behaviour. This may be problematic given (a) existing findings implicating chronic nicotine exposure in alteration of response inhibition (e.g. Luijten et al., 2011) and impulsive choice (e.g. Kolokotroni et al., 2014), and (b) increasing agreement that stimulant dependence may lead to more severe neurological impairment than cannabis dependence (Bonomo et al., 2019; Van Holst and Schilt, 2011).

A dimension of impulsive behaviour now recognised to be distinguishable from response inhibition is reflection impulsivity – the tendency to gather and evaluate information prior to decision-making (Kagan, 1966) – measured using the information sampling task (IST). On the IST respondents make correct or incorrect judgements under varying levels of uncertainty, with a lower probability of being correct (p(correct)) at point of decision-making being indicative of disrupted reflection impulsivity (Clark et al., 2006, 2009). Using this paradigm, Clark et al. (2009) found those who smoke both cannabis and cigarettes display impaired reflection impulsivity, evidenced through sampling less information and lower p(correct), compared with non-users of either drug. Impaired reflection impulsivity on the IST has also been demonstrated in adolescent cannabis only smokers relative to controls (Solowij et al., 2012). At no point, however, have cigarette only smokers been compared on the IST relative to NS, cannabis only smokers and smokers of both cannabis and cigarettes. This is an important next step needed in order to understand the effects of cigarette smoking and cannabis smoking on this form of impulsivity.

In summary, given the high prevalence rates of co-morbid cannabis-cigarette smoking, it is critical to further understand the role of impulsivity when these behaviours are performed both in isolation and in combination. Past research that has attempted to establish the role of impulsivity in cannabis use has been limited by a failure to consistently control for co-morbid cigarette smoking. The present study builds on previous research by utilising a multivariate approach to understanding the role of impulsivity in cannabis use. Differences between cigarette and cannabis smokers on both trait and behavioural impulsivity were assessed using a sample that included NS, cigarette only, cannabis only and cannabis plus cigarette smokers. Importantly, both cannabis groups in this study used tobacco in their joints ensuring high ecological validity, accurately representing how cannabis is smoked in Europe. Critically, this design enabled the first step towards disentangling cannabis smoking from cigarette smoking with respect to the unique relationships different facets of impulsivity may have with each of these behaviours.

## Methods

### Participants

#### Trait impulsivity participant sample

Trait impulsivity questionnaire data were gathered from a series of studies investigating the role of impulsivity in drug use with a total sample of 224. There were 44 NS (age *M *= 24.73, SD = 4.57; Male = 57%), 76 cigarette only smokers (age *M* = 23.55, SD = 5.75; Male = 55%), 47 cannabis only smokers (age *M* = 21.98, SD = 4.59; Male = 64%) and 58 cannabis plus cigarette smokers (age *M* = 22.62, SD = 3.37; Male = 74%). Individuals were recruited throughout the UK using opportunity and snowball sampling; all participants provided written informed consent. This study was approved by the Research Ethics Committees of Leeds Beckett University and the University of Leeds (approval reference: 15-0095) and was conducted according to the World Medical Association Declaration of Helsinki. Participants were excluded from participating if they were non-native English speakers; reported a history of psychiatric illness as defined in the *Diagnostic and Statistical Manual of Mental Disorders* (DSM-5; American Psychiatric Association, 2013), including disorders where impulsivity is a hallmark feature, e.g. attention deficit hyperactivity disorder; had a brain injury, or severe head trauma; were trying to abstain from smoking tobacco or cannabis; or were dependent on any drugs (including alcohol) other than nicotine or cannabis.

Participants were assigned to groups according to drug use frequency and duration of use, an approach that is common in the smoking literature (e.g. De Leon et al., 2003). In order to reach criteria for a cannabis smoker, individuals needed to have smoked cannabis once or more a week for the last 6 months and score ⩾13 on the Cannabis Use Disorders Identification Test – Revised (CUDIT-R), indicative of cannabis dependence (Adamson et al., 2010). Cannabis smokers who also smoked cigarettes daily and had done so for the last 6 months were classed as cannabis plus cigarette smokers; those who reported being non-smokers of cigarettes were classed as cannabis only smokers. Cigarette smokers had to have smoked tobacco cigarettes daily for the last 6 months and were selected in order to match cannabis plus cigarette smokers on frequency of cigarette smoking (this ranged between 3 and 20 cigarettes per day). NS reported no use of tobacco or any illicit substances and no current/past dependence on any substance.

#### Behavioural impulsivity participant sample

Eighty-seven participants from the total sample (*N* = 224) also completed behavioural impulsivity tasks. This sample comprised 30 NS (age *M* = 24.43, SD = 4.15; Male = 47%), 29 cigarette only smokers (age *M* = 24.97, SD = 7.21; Male = 52%), 13 cannabis only smokers (age *M* = 22.23, SD = 2.83; Male = 46%), and 15 cannabis plus cigarette smokers (age *M* = 24.00, SD = 4.36; Male = 80%).

### Measures

#### Smoking behaviour and craving

Nicotine dependence was assessed using the Fagerström Test for Nicotine Dependence (FTND; Heatherton et al., 1991). Cannabis dependence was assessed using the CUDIT-R (Adamson et al., 2010). To assess smoking satiety on arrival at the behavioural session participants also self-reported recent drug use, drug craving and undertook expired carbon monoxide (CO) assessment with a Micro^+^ Smokerlyzer (Bedfont, Maidstone, UK). Cigarette craving was measured using the Questionnaire of Smoking Urges – Brief (QSU-Brief; Cox et al., 2001), which consists of two sub-factors: positive (Cronbach’s α = .95) and negative craving (α = .94). Cronbach’s α for the QSU-Brief total score was .96. Cannabis craving was measured using the Marijuana Craving Questionnaire – Short Form (MCQ-SF; Heishman et al., 2009), which consists of four sub-factors: compulsivity, emotionality, expectancy and purposefulness craving. Cronbach’s α for sub-factors ranged from .49 to .77 and the MCQ-SF total score α was .77.

#### Trait impulsivity

Trait impulsivity was assessed using the Barratt Impulsiveness Scale Version 11 (BIS-11; Patton and Stanford, 1995), which consists of three sub-factors: attentional, motor, and non-planning impulsiveness. Cronbach’s α for BIS-11 sub-factors ranged from .70 to .75 and α for the BIS-11 total score was .84.

### Behavioural impulsivity

#### Information sampling task (IST)

The IST (Clark et al., 2006) was used to assess reflection impulsivity. The IST was composed of a single practice trial followed by 10 trials in each of two conditions: a fixed win (FW) condition and a decreasing win (DW) condition. On each trial a 5 × 5 matrix of grey boxes was presented, with a panel containing two boxes (one red and one blue) directly below. Selecting a grey box caused it to open immediately (for the duration of the trial) revealing a red or blue box. Respondents chose how many grey boxes to open, before deciding which of the two colours underlaid the majority in the matrix. Upon indicating a decision, any unopened boxes opened and a feedback message ‘Correct! You have won (x) points’ or ‘Wrong! You have lost 100 points’ was presented for 2 s. In the FW condition, the subject won 100 points for making a correct decision, regardless of the number of boxes opened. In the DW condition, the amount available to win started at 250 points decreasing by 10 points with each box opened. An incorrect decision resulted in a 100-point deduction from the total points scored. A variable delay (1s minimum) between trial onsets was used ensuring a minimum intertrial interval of 30 s. Mean probability of being correct at the point of decision, p(correct), is the primary index of reflection impulsivity; lower p(correct) is indicative of greater impulsivity. P(correct) is calculated as follows (equation (1); Clark et al., 2006):

(1) $$P(correct)=\frac{\sum^{z}k=A\left(\frac{z}{k}\right)}{2^{z}}$$

where *Z* = 25 – (number of boxes opened), and *A* = 13 – (number of boxes opened of the chosen colour). For example, if a decision is made towards red after opening 10 boxes (8 red, 2 blue), then *Z* = 25 – 10 = 15, *A* = 13 – 8 = 5, and p(correct) = [15!/(5! × 10!) + 15!/(6! × 9!) + . . . + 15!/(15! × 0!)]/2^15^ = 0.94 (example taken from Clark et al., 2006: 518).

Secondary dependent variables include mean number of boxes opened, mean incorrect judgements, mean latency of box opening, and total points won.

#### Stop-signal task (SST)

The SST ran through STOP-IT software and was used to evaluate response inhibition (Logan et al., 1997; Verbruggen et al., 2008). The present SST ran three blocks of trials (63 trials per block; 189 total), preceded by a practice block (15 trials). Trials began with a central fixation point presented for 500 ms, immediately followed by either a square or a circle for 1250 ms. Participants had to respond (on 75% of trials) by pressing left or right on the keyboard on presentation of a square or circle respectively. Randomly (on 25% of trials), participants were presented with an auditory stop-signal that occurred after varying delays following presentation of a square/circle. On receipt of this signal, participants had to withhold responding for that trial. Stop-signals were presented for 250 ms through external speakers and were randomly preceded by an equal number of squares and circles per block. The stop-signal delay (SSD) was initially fixed at 250 ms and subsequently titrated by 50 ms contingent on the previous stop-signal response using a staircase-tracking algorithm. This procedure adjusts the SSD until successful inhibition is achieved on 50% of stop-signal trials, allowing for estimation of the stop-signal reaction time (SSRT). SSRT was calculated by subtracting SSD from mean primary task reaction time. SSRT is the primary dependent variable; longer SSRT is indicative of poor response inhibition. Secondary dependent variables include: SSD, probability of responding on stop-signal trials, percentage of correct and missed responses, commission error reaction time, and correct response reaction time.

### Procedure

All participants initially completed a questionnaire pack including measures of drug use, drug dependence, and trait impulsivity. The behavioural impulsivity sample then attended a test session where behavioural impulsivity tasks were completed. Individuals were asked to refrain from consuming >2 units of alcohol in the 24 h before the session or any caffeine in the 2 h before the session, to avoid residual and acute effects of these substances on performance. Cannabis groups were permitted to smoke cannabis *ad-libitum* but were asked to avoid using cannabis 3 h before the session to avoid acute intoxication (Meyer and Quenzer, 2013). Cigarette smoking before the session was *ad-libitum*. Smoking satiety was verified through self-report of last drug use, drug craving and expired CO at arrival to the session.

### Statistical analysis

Data were analysed using SPSS Version 24 (Armonk, NY: IBM Corp.). Prior to inferential analyses, normality was assessed using the Shapiro–Wilk test and homogeneity of variance was assessed using Levene’s test. Between-subject *t*-tests were used to compare groups on smoking behaviour and craving scores, and to verify whether participants who completed the behavioural impulsivity measures differed significantly in age, smoking behaviour and trait impulsivity scores from those who completed only the trait impulsivity measures. Variables violating normality were assessed using the non-parametric Mann–Whitney U test. Pearson’s chi-square test was used to compare sex ratio across groups and samples.

Two (cigarette smoking status: cigarette smokers vs non-cigarette smokers) × two (cannabis smoking status: cannabis smokers vs non-cannabis smokers) between-subject ANOVAs were used to compare groups on age, expired CO, trait and behavioural impulsivity scores. Simple effects analyses with Bonferroni adjustment for multiple comparisons were used to explore significant interaction effects. On the SST, SSRT can only be reliably estimated when the mean probability of responding on stop-signal trials is close to 50% (Verbruggen et al., 2019). Consistent with this recommendation and previous work (Congdon et al., 2012), all individuals who inhibited less than 40% or greater than 60% of stop-signal trials (reflecting values falling one standard deviation outside of the mean probability of responding on stop-signal trials), were therefore omitted from analysis. Ten participants (six NS, two cigarette only, one cannabis only, one cannabis plus cigarette smoker) were excluded from SST analysis. Pearson’s correlations were used to explore relationships between trait impulsivity, drug use and drug dependence, independently by drug group. Alpha (two-tailed) was set at *p* < .05.

## Results

### Trait impulsivity participant sample characteristics

As shown in Table 1, there was no significant main effect of cigarette smoking status or interaction with cannabis status on age. However, a significant main effect of cannabis smoking status revealed non-cannabis smokers were significantly older than cannabis smokers. Sex ratio did not differ significantly across groups.

**Table 1.** Trait impulsivity participant sample demographics and smoking behaviour (*N* = 224).

	NS		CAN NON-CIG		CIG		CAN CIG		Test statistic	*P* value	Effect size
*N*	44		47		76		58		–	–	–
Age years **	24.73	(4.57)	21.98	(4.59)	23.55	(5.75)	22.62	(3.37)	_Cannabis main effect_ *F*_(1, 221)_ = 8.05 _Cigarette main effect_ *F*_(1, 221)_ < 1 _Cannabis*cigarette interaction_ *F*_(1, 221)_ = 1.96	.005 .682 .163	η_p_^2^ = .04 η_p_^2^ = .00 η_p_^2^ = .01
Sex M:F	25:19	–	30:17	–	42:34	–	43:15	–	*χ*^2^_(3)_ = 5.67	.130	–
Cigarettes smoked per day	–	–	–	–	10.66	(4.93)	9.46	(5.12)	*t*_(128)_ = 1.35	.179	*d* = .24
Years smoking tobacco	–	–	–	–	5.89	(5.68)	5.30	(3.54)	*t*_(127)_ < 1	.494	*d* = .13
FTND	–	–	–	–	3.19	(2.23)	2.89	(2.09)	*t*_(128)_ < 1	.440	*d* = .14
Proportion of time using tobacco in joints (%)	–	–	73.41	(32.67)	–	–	90.25*	(12.50)	*U* = 1026.50	.030	*r* = .21
Days per week smoking cannabis	–	–	5.11	(1.87)	–	–	5.51	(1.78)	*t*_(101)_ = 1.11	.270	*d* = .22
Days per month smoking cannabis	–	–	21.09	(8.03)	–	–	23.44	(7.28)	*t*_(101)_ = 1.56	.123	*d* = .31
Years smoking cannabis	–	–	4.62	(3.75)	–	–	5.71	(4.08)	*t*_(101)_ = 1.40	.164	*d* = .28
CUDIT-R	–	–	16.72	(3.91)	–	–	18.81*	(4.58)	*t*_(101)_ = 2.45	.016	*d* = .49

Data presented as mean (SD) or frequency. NS: non-smoker of cigarettes and cannabis; CAN NON-CIG: cannabis only smoker; CIG: cigarette only smoker; CAN CIG: cannabis plus cigarette smoker; M: male, F: female; FTND: Fagerström Test for Nicotine Dependence; CUDIT-R: Cannabis Use Disorders Identification Test –Revised. *F*-values presented refer to 2 (cannabis status) × 2 (cigarette status) ANOVA statistics. Asterisks indicate level of significance: *p < .05, **p < .01.

Importantly the two cannabis groups reported comparable levels of cannabis smoking frequency and years smoking cannabis. Despite comparable cannabis use, the cannabis plus cigarette group reported significantly greater cannabis dependence than the cannabis only smokers. Cannabis plus cigarette smokers also reported using tobacco in joints significantly more than cannabis only smokers. Cigarette only smokers did not differ significantly from the cannabis plus cigarette group on daily cigarette smoking, years smoking tobacco, or levels of nicotine dependence.

### Trait impulsivity

**Figure 1.**
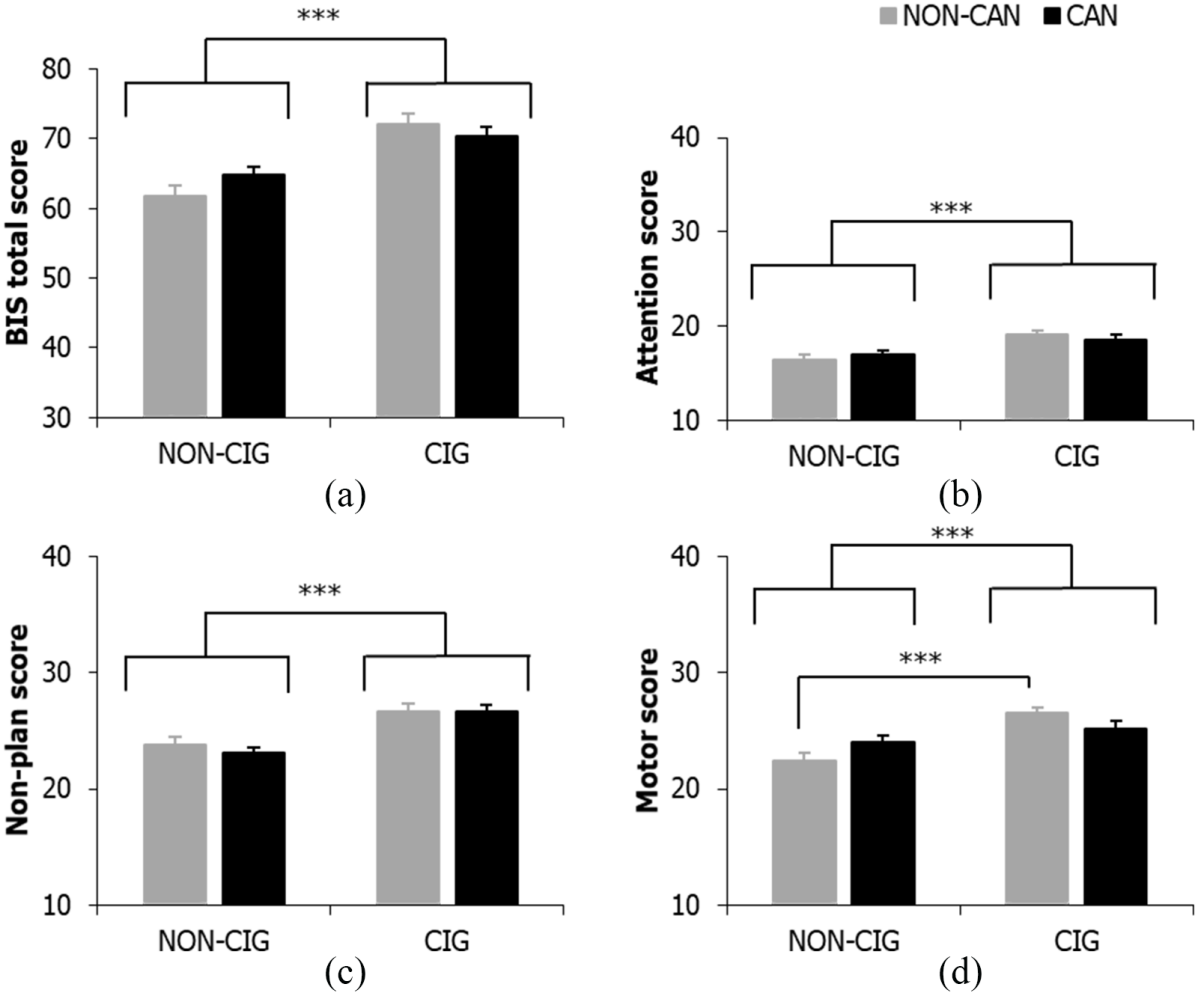
Main effect of cigarette status for (a) BIS-11 total, (b) attention and (c) non-planning subscales; (d) cigarette × cannabis status interaction for BIS-11 motor impulsivity score. Bars represent mean ± SEM. Asterisks indicate level of significance: ***p < .001.

There were significant main effects for cigarette smoking status on BIS-11 total scores (*F*_(1, 220)_ = 32.76, *p* < .001, η_p_^2^ = .13), along with the attention (*F*_(1, 220)_ = 15.63, *p* < .001, η_p_^2^ = .07) and non-planning subscales (*F*_(1, 220)_ = 27.95, *p* < .001, η_p_^2^ = .11), such that individuals who smoked cigarettes scored significantly higher than those who did not smoke cigarettes (Figure 1(a) to (c)). No significant effects of cannabis smoking or interactions were found (all *F*_(1, 220)_ < 2.27, *p* ⩾ .101, η_p_^2^ ⩽ .01).

For the BIS-11 motor impulsivity subscale, there was again no significant main effect of cannabis (*F* < 1, *p* = .773, η_p_^2^ < .001), but a significant main effect of cigarette smoking (*F*_(1, 220)_ = 19.14, *p* < .001, η_p_^2^ = .08), this time qualified by a significant cigarette smoking × cannabis smoking interaction (*F*_(1, 220)_ = 5.92, *p* = .016, η_p_^2^ = .03; see Figure 1d). Cigarette smoking was again associated with higher levels of impulsivity, but simple effects analyses showed that this effect was seen in NS (*p *< .001), but not smokers (*p* = .178) of cannabis. Motor impulsivity did not differ as a function of cannabis smoking in either cigarette smokers (*p* = .095) or non-cigarette smokers (*p *= .078).

### Associations between trait impulsivity, cigarette use, and nicotine dependence

As shown in Table 2, in cigarette only smokers, higher total, motor and non-planning impulsivity were associated with increased daily smoking of cigarettes and greater levels of nicotine dependence. A negative association was revealed between total impulsivity and years smoking tobacco. In contrast, in those who smoked cannabis and cigarettes, greater total impulsivity was associated with increased daily cigarette smoking, while greater attentional impulsivity was associated with increased levels of nicotine dependence.

**Table 2.** Pearson’s correlations between trait impulsivity and tobacco use and dependence by group.

CIG	BIS-11			
	Total	Attention	Motor	Non-plan
Cigarettes smoked per day (*n* = 73)	.27*	.05	.26*	.32**
Years smoking tobacco (*n* = 72)	−.23*	−.19	−.16	−.22
FTND (*n* = 73)	.33**	.19	.27*	.34**
CAN CIG				
Cigarettes smoked per day (*n* = 56)	.28*	.21	.25	.21
Years smoking tobacco (*n* = 57)	.17	.09	.13	.19
FTND (*n* = 57)	.17	.28*	.10	.07

Data represent Pearson’s *r* correlation coefficients. BIS-11: Barratt Impulsiveness Scale Version 11; CIG: cigarette only; CAN CIG: cannabis plus cigarette smoker. Asterisks indicate level of significance: *p <.05, **p < .01.

### Associations between trait impulsivity, cannabis use, and cannabis dependence

No significant relationships were found between trait impulsivity and either cannabis dependence or frequency of cannabis use in either cannabis group, with the exception of greater motor impulsiveness, which was associated with fewer days per week smoking cannabis in the cannabis plus cigarette smoking group (see Table 3).

**Table 3.** Pearson’s correlations between trait impulsivity and cannabis use and dependence by group.

CAN NON-CIG	BIS-11			
	Total	Attention	Motor	Non-plan
Days per week smoking cannabis (*n* = 46)	−.18	−.11	−.03	−.28
Years smoking cannabis (*n* = 46)	.04	−.08	.10	.06
CUDIT-R (*n* = 46)	.04	.26	−.03	−.08
CAN CIG				
Days per week smoking cannabis (*n* = 57)	−.17	.00	−.30*	−.09
Years smoking cannabis (*n* = 57)	.15	.11	.09	.17
CUDIT-R (*n* = 57)	.15	.05	.09	.21

Data represent Pearson’s *r* correlation coefficients. BIS-11: Barratt Impulsiveness Scale Version 11; CAN NON-CIG: cannabis only smoker; CAN CIG: cannabis plus cigarette smoker. Asterisks indicate level of significance: *p < .05, **p < .01.

### Behavioural impulsivity participant sample characteristics

There was no significant main effect of cigarette smoking, cannabis smoking or their interaction on age, and sex ratio was equal across groups (see Table 4).

**Table 4.** Behavioural impulsivity participant sample demographics and smoking behaviour (*N* = 87).

	NS		CAN NON–CIG		CIG		CAN CIG		Test statistic	*P* value	Effect size
*N*	30		13		29		15		–	–	–
Age years	24.43	(4.15)	22.23	(2.83)	24.97	(7.21)	24	(4.36)	Cannabis main effect *F*(1, 83) = 1.70 Cigarette main effect *F*(1, 83) < 1 Cannabis*cigarette interaction *F*(1, 83) < 1	.196 .346 .612	ηp^2^ = .02 ηp^2^ = .01 ηp^2^ = .00
Sex M:F	14:16		6:07		15:14		12:03		*χ*^2^(3) = 5.12	.172	–
Cigarettes smoked per day	–	–	–	–	9.88	(4.83)	11.83	(6.01)	*t*(42) = 1.17	.249	*d* = .36
Years smoking tobacco	–	–	–	–	6.52	(6.13)	6.03	(4.72)	*t*(42) < 1	.791	*d* = .09
FTND	–	–	–	–	3.10	(2.35)	3.53	(1.64)	*t*(42) < 1	.531	*d* = .22
Proportion of time using tobacco in joints (%)	–	–	84.17	(18.89)	–	–	87.80	(9.54)	*U* = 96.50	.972	*r* = .01
Days per week smoking cannabis	–	–	5.00	(1.91)	–	–	5.40	(1.97)	*t*(26) < 1	.591	*d* = .21
Days per month smoking cannabis	–	–	21.42	(8.01)	–	–	23.30	(8.21)	*t*(26) < 1	.547	*d* = .23
Years smoking cannabis	–	–	4.46	(2.50)	–	–	7.01	(5.53)	*t*(20.06) = 1.61	.124	*d* = .64
CUDIT–R	–	–	16.38	(4.33)	–	–	17.33	(3.48)	*t*(26) < 1	.526	*d* = .24
CO (parts per million) ***	2.10	(0.88)	3.42	(1.24)	10.34	(7.71)	11.80	(4.31)	Cannabis main effect *F*(1, 130) = 3.76 Cigarette main effect *F*(1, 130) = 50.83 Cannabis*cigarette interaction *F*(1, 130) < 1	.055 <.001 .466	ηp^2^ = .03 ηp^2^ = .28 ηp^2^ = .00
Time (min) of last cigarette before session	–	–	–	–	30.00	(57.50)	20.00	(47.50)	*U* = 214.50	.946	*r* = .01
Time (hours) of last cannabis use before session	–	–	14:00	(4.25)	–	–	12:00	(4.50)	*U* = 56.00	.055	*r* = .36
QSU-Brief total craving	–	–	–	–	41.38	(27.97)	32.60	(21.03)	*t*(42) = 1.07	.292	*d* = .36
QSU-Brief positive craving	–	–	–	–	26.07	(15.11)	23.00	(14.03)	*t*(42) = 1.42	.165	*d* = .21
QSU-Brief negative craving	–	–	–	–	15.31	(14.37)	9.60	(8.38)	*t*(42) < 1	.517	*d* = .50
MCQ-SF total craving	–	–	43.69	(11.22)	–	–	45.29	(8.96)	*t*(25) < 1	.686	*d* = .16
Compulsivity craving	–	–	6.08	(3.52)	–	–	6.80	(2.81)	*t*(26) < 1	.551	*d* = .23
Emotionality craving	–	–	10.08	(3.17)	–	–	10.07	(3.85)	*t*(26) < 1	.994	*d* = .00
Expectancy craving	–	–	13.62	(3.91)	–	–	13.47	(3.29)	*t*(26) < 1	.914	*d* = .04
Purposefulness craving	–	–	13.92	(4.17)	–	–	15.27	(4.04)	*t*(26) < 1	.395	*d* = .33

Data presented as mean (SD) or frequency. NS: non–smoker of cigarettes and cannabis; CAN NON–CIG: cannabis only smoker; CIG: cigarette only smoker; CAN CIG: cannabis plus cigarette smoker; M: male, F: female; FTND: Fagerström Test for Nicotine Dependence; QSU-Brief: Questionnaire of Smoking Urges; MCQ-SF: Marijuana Craving Questionnaire; CUDIT–R: Cannabis Use Disorders Identification Test – Revised; CO: carbon monoxide. *F*–values presented refer to 2 (cannabis status) × 2 (cigarette status) ANOVA statistics. Asterisks indicate level of significance: ***p < .001.

Cannabis groups did not differ significantly on weekly or monthly cannabis use, years smoking cannabis, cannabis dependence, use of tobacco in joints, or time (hours) since last cannabis use prior to the session (Table 4). The two cannabis groups also reported comparable cannabis craving on (total and subscales of) the MCQ-SF on arrival at the session.

Cigarette only and cannabis plus cigarette groups did not differ significantly on cigarettes smoked per day, years smoking tobacco, nicotine dependence, or time (min) since last cigarette prior to the session (Table 4). The two cigarette groups also reported comparable cigarette craving on (total and subscales of) the QSU-Brief on arrival at the session.

A significant main effect of cigarette smoking status for expired CO levels was found (Table 4), indicating that cigarette smokers had significantly greater CO levels compared with non-smokers of cigarettes at arrival to the session. No significant main effect of cannabis smoking status or a cannabis × cigarette smoking status interaction were revealed.

### Comparison of trait-only participants and behavioural impulsivity sample

The behavioural impulsivity sample did not differ significantly from participants who had only completed the trait impulsivity measures in terms of age, sex ratio, drug use or trait impulsivity scores (see Table 5).

**Table 5.** Comparison of trait–only participants and behavioural sample on demographics, drug use, and trait impulsivity.

	Trait–only participants		Behavioural sample		Test statistic	*P* value	Effect size
*N*	137		87		–	–	–
Age	23.50	(5.05)	24.21	(5.27)	*t*_(222)_ < 1	.319	*d* = .14
Sex M:F	81:56	–	47:40	–	*χ*^2^_(1)_ < 1	.490	–
Cigarettes smoked per day	9.93	(5.07)	10.55	(5.28)	*t*_(102)_ < 1	.546	*d* = .12
Years smoking tobacco	6.13	(5.09)	6.35	(5.63)	*t*_(102)_ < 1	.830	*d* = .04
FTND	3.20	(2.16)	3.25	(2.13)	*t*_(102)_ < 1	.907	*d* = .02
Proportion of time using tobacco in joints (%)	80.32	(27.51)	85.82	(14.21)	*U* = 1068.50	.944	*r* = .01
Days per week smoking cannabis	5.12	(1.90)	5.21	(1.92)	*t*_(103)_ < 1	.817	*d* = .05
Days per month smoking cannabis	21.49	(8.16)	22.43	(8.02)	*t*_(103)_ < 1	.603	*d* = .12
Years smoking cannabis	5.39	(4.15)	5.83	(4.51)	*t*_(103)_ < 1	.638	*d* = .10
CUDIT–R	17.12	(4.19)	16.89	(3.85)	*t*_(103)_ < 1	.805	*d* = .06
BIS–11 total	66.60	(10.84)	66.63	(10.70)	*t*_(222)_ < 1	.982	*d* = .00
Attention	17.47	(4.08)	17.30	(4.12)	*t*_(222)_ < 1	.765	*d* = .04
Motor	24.36	(4.62)	24.39	(4.51)	*t*_(222)_ < 1	.967	*d* = .01
Non–plan	24.77	(4.80)	24.94	(4.82)	*t*_(222)_ <1	.790	*d* = .04

Data presented as mean (SD) or frequency. M: male, F: female; FTND: Fagerström Test for Nicotine Dependence; CUDIT–R: Cannabis Use Disorders Identification Test – Revised; BIS–11: Barratt Impulsiveness Scale Version 11.

### Reflection impulsivity

#### P(correct)

**Figure 2.**
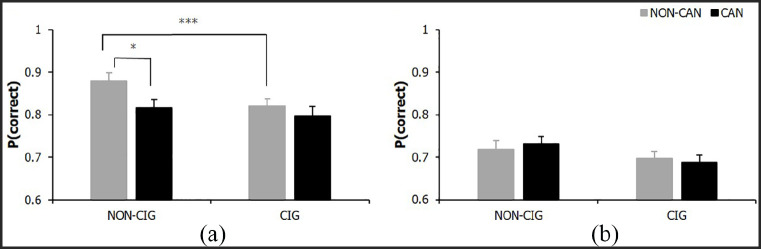
P(correct) for (a) FW cigarette × cannabis status interaction and (b) DW descriptive statistics. Bars represent mean ±SEM. Asterisks indicate level of significance: *p < .05, ***p < .001.

For FW trials, no significant main effects for cigarette or cannabis smoking status (both *F* < 1, *p* ⩾ .370, η_p_ ⩽ .01), were revealed on p(correct); there was, however, a significant cannabis × cigarette smoking status interaction (*F*_(1, 83)_ = 9.14, *p* = .003, η_p_ = .10). The three smoking groups had similar p(correct) scores that were lower than NS of either substance; see Figure 2a. Simple effects analyses showed that cigarette only (*p* = .001) and cannabis only (*p* = .011) smokers had significantly lower p(correct) scores compared with NS. However, using both cigarettes and cannabis together did not lead to significantly further reductions in p(correct) scores relative to using either cigarettes (*p* = .101) or cannabis (*p* = .202) alone.

For DW trials there were no significant main or interaction effects for p(correct) (all *F*_(1, 83)_ ⩽ 2.58, *p* ⩾ .112, η_p_ = .03; see Figure 2b).

#### Boxes opened

**Figure 3.**
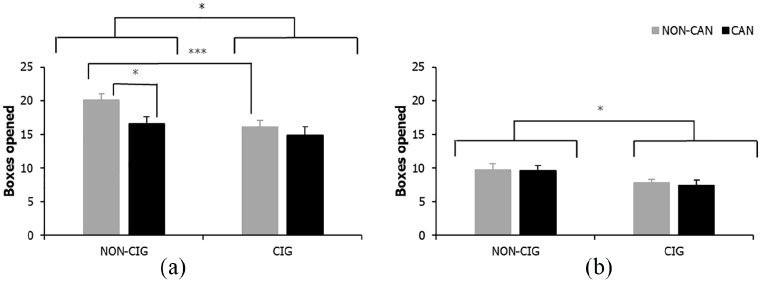
Number of boxes opened for (a) FW cigarette × cannabis status interaction and (b) DW main effect of cigarette status. Bars represent mean ± SEM. Asterisks indicate level of significance: *p < .05, ***p <.001.

For FW number of boxes opened, there was no significant main effect of cannabis smoking status (*F* < 1, *p* = .541, η_p_ = .01), but a significant main effect of cigarette smoking status (*F*_(1, 83)_ = 4.26, *p* = .042, η_p_ = .05), and a significant cigarette smoking × cannabis smoking status interaction (*F*_(1, 83)_ = 5.52, *p* = .021, η_p_ = .06). Simple effects analyses revealed cigarette only (*p* < .001) and cannabis only (*p* = .043) smokers opened significantly fewer boxes compared with NS of either substance. However, using both cigarettes and cannabis was not associated with a further reduction in box opening, relative to using either cigarettes (*p* = .213) or cannabis (*p* = .863) alone, see Figure 3a.

For DW number of boxes opened there was a significant main effect of cigarette smoking status (*F*_(1, 83)_ = 4.30, *p* = .041, η_p_ = .05), such that smokers of cigarettes opened significantly fewer boxes compared with individuals who did not smoke cigarettes (Figure 3b). There was no significant main effect for cannabis smoking status or cigarette × cannabis status interaction on the number of boxes opened (both *F* < 1, *p *⩾ .513, η_p_ ⩽ .01).

#### Incorrect judgements

**Figure 4.**
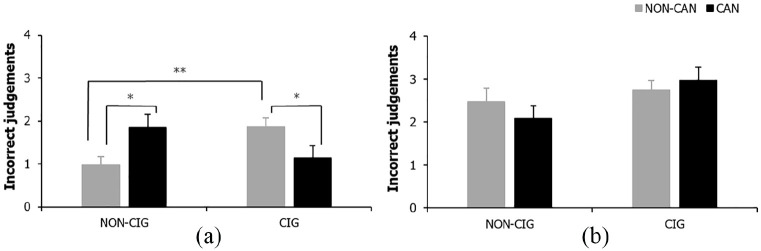
Incorrect judgements for (a) FW cigarette × cannabis status interaction and (b) DW descriptive statistics. Bars represent mean ±SEM. Asterisks indicate level of significance: *p < .05, **p < .01.

For FW incorrect judgements there were no significant main effects for cigarette or cannabis smoking status (both *F* < 1, *p* ⩾ .726, η_p_ ⩽ .00); however there was a significant cigarette × cannabis smoking status interaction(*F*_(1, 83)_ = 9.59, *p* = .003, η_p_ = .10). Simple effects analyses showed cigarette only (*p* = .003) and cannabis only (*p *= .021) smokers made significantly more incorrect judgements compared with NS of either substance. In this case, cigarette only (*p* = .046) but not cannabis only smokers (*p *> .999) also made significantly more incorrect judgements relative to users of both drugs, see Figure 4a.

For DW incorrect judgements there were no significant main or interaction effects (all *F*_(1, 83)_ ⩽ 2.74, *p* ⩾ .102, η_p_ ⩽ .03; see Figure 4b).

#### Latency of box opening

A significant main effect of cigarette smoking status on FW latency of box opening (*F*_(1, 83)_ = 4.53, *p* = .036, η_p_ = .05) revealed cigarette smokers took longer to open boxes than non-cigarette smokers

**Figure 5.**
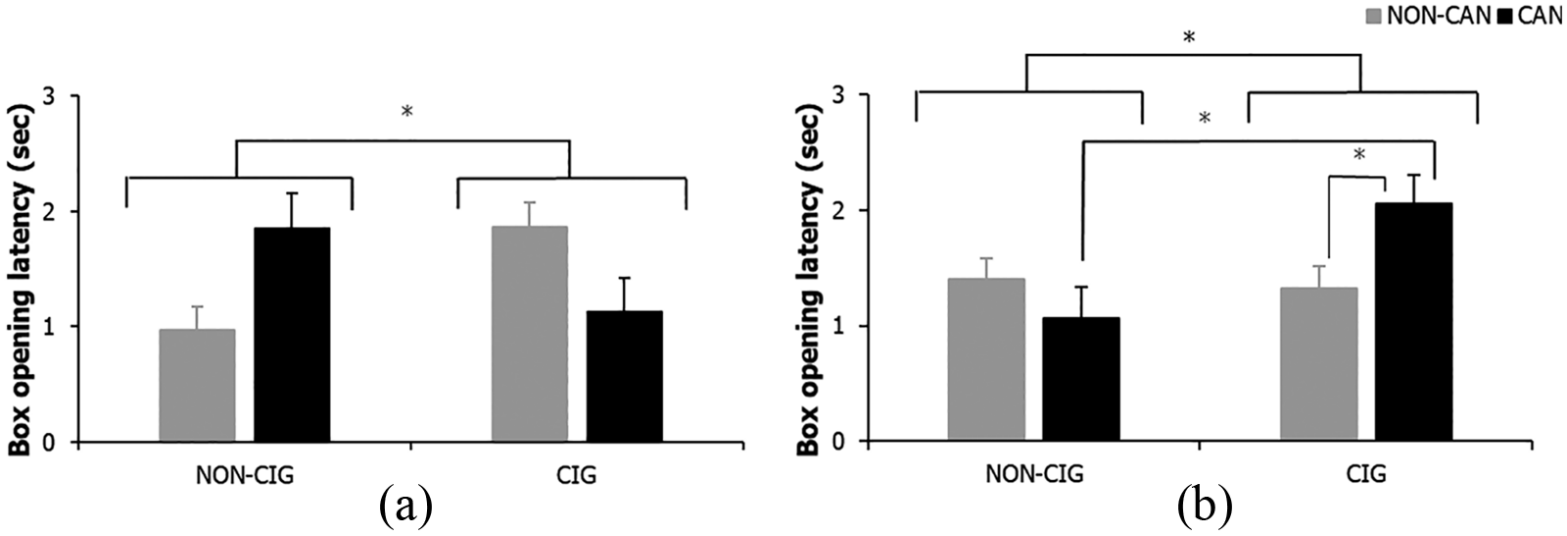
Latency of box opening for (a) FW main effect of cigarette status and (b) DW cigarette × cannabis status interaction. Bars represent mean ± SEM. Asterisks indicate level of significance: *p < .05.

There was also a significant main effect of cigarette smoking for DW latency of box opening (*F*_(1, 83)_ = 4.08, *p* = .047, η_p_ = .05), this time qualified by a significant cigarette smoking × cannabis smoking status interaction (*F*_(1, 83)_ = 5.47, *p* = .022, η_p_ = .06). Simple effects analyses showed cigarette only (*p* = .778) and cannabis only (*p* = .300) smokers did not differ significantly from NS on time spent opening boxes. However, cannabis plus cigarette smokers spent significantly longer opening boxes than cigarette only (*p* = .024) and cannabis only smokers (*p* = .010), see Figure 5b. There was no significant main effect of cannabis status (*F* < 1, *p* = .408, η_p_ = .01)

**Figure 6.**
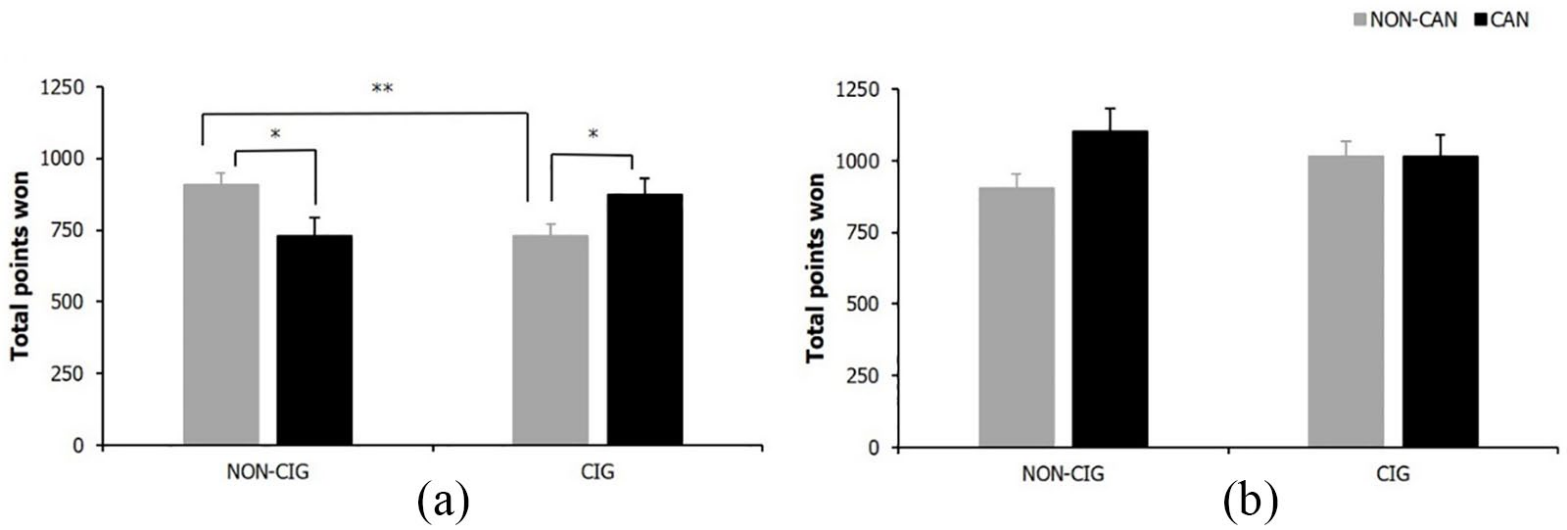
Total points won for (a) FW cigarette × cannabis status interaction and (b) DW descriptive statistics. Bars represent mean ±SEM. Asterisks indicate level of significance: *p < .05, **p < .01.

***Total points won***. For FW total points won there were no significant main effects for cigarette or cannabis smoking status (both *F* < 1, *p* ⩾ .726, ηp ⩽ .00); however, there was a significant cigarette × cannabis smoking status interaction (*F*(1, 83) = 9.59, *p* = .003, ηp = .10). Simple effects analyses showed cigarette only (*p* = .003) and cannabis only (*p* = .021) smokers won significantly fewer total points than NS. Cigarette only (*p* = .046) but not cannabis only smokers (*p* > .999), also won significantly fewer points than cannabis plus cigarette smokers, see Figure 6a.

In contrast, for DW trials there was no significant main or interaction effects on total points won (all *F*(1, 83) ⩽ 2.26, *p* ⩾ .136, ηp ⩽ .03; see Figure 6b).

### Response inhibition

There were no significant main or interaction effects on SSRT or any secondary dependent variables of the SST (all *F*_(1, 73)_ ⩽ 2.86, *p* ⩾ .095, η_p_ ⩽ .04); see Table 6 for SST dependent variable descriptive statistics.

**Table 6.** Descriptive statistics for stop-signal task group comparisons.

	NS		CAN NON-CIG		CIG		CAN CIG	
*N* ^a^	24		12		27		14	
SSRT (ms)	277.70	(32.07)	282.43	(32.28)	260.91	(63.60)	289.52	(71.51)
SSD (ms)	275.42	(114.01)	325.78	(189.86)	390.66	(187.77)	309.32	(147.42)
P(respond|signal) (%)	48.23	(4.71)	47.43	(4.93)	47.35	(5.84)	48.05	(6.45)
Correct responses (%)	98.39	(1.94)	97.00	(3.88)	96.90	(5.02)	97.59	(2.04)
Missed responses (%)	0.94	(1.87)	2.12	(3.92)	1.46	(2.16)	1.36	(2.30)
Commission error RT (ms)	477.29	(89.38)	522.53	(141.06)	569.85	(133.50)	523.80	(112.58)
Correct response RT (ms)	553.79	(111.75)	609.37	(174.19)	651.88	(154.29)	599.86	(157.30)

Data presented as mean (SD). NS: non-smoker of cigarettes and cannabis; CAN NON-CIG: cannabis only smoker; CIG: cigarette only smoker; CAN CIG: cannabis plus cigarette smoker; SSRT: stop-signal reaction time; SSD: stop-signal delay; P(respond|signal): probability of responding on stop-signal trials; ms: milliseconds; RT: reaction time.

a Ten participants (six NS, two cigarette only, one cannabis only, one cannabis plus cigarette smokers) were excluded from the stop-signal task analysis due to inability to accurately estimate SSRT.

## Discussion

It is unclear from existing research how the role of impulsivity may differ across cannabis only, cigarette only and cigarette plus cannabis users, and to what extent cigarette smoking may account for the heightened impulsivity previously found in cannabis users. To explore this, the present study assessed the effects of cigarette and cannabis smoking on trait and behavioural impulsivity across a sample of NS, cigarette only, cannabis only, and cigarette plus cannabis smokers. Findings demonstrated clear differences between these groups on elements of trait and behavioural impulsivity, and how these relate to key cigarette and cannabis use outcomes, suggesting that unique aspects of impulsivity may be differentially related to cannabis and cigarette smoking. Each of these findings will be discussed in more detail below.

Total, attention and non-planning trait impulsivity were significantly higher in cigarette smokers (cannabis and non-cannabis users) compared with individuals who did not smoke cigarettes. Elevated trait motor impulsiveness was also observed in cigarette smokers, however, on this component of impulsivity the effect of cigarette smoking was specific to those who did not also use cannabis. In contrast, trait impulsivity appeared to be unrelated to cannabis use. The present findings stand in opposition to previous research demonstrating greater trait motor and non-planning impulsivity in cannabis only smokers compared with NS (Gilman et al., 2015), instead suggesting that impulsive tendencies may be especially prevalent in cigarette smokers compared with NS. The present findings are however consistent with previous work demonstrating elevated trait impulsivity on the BIS-11 in both cigarette only and cannabis plus cigarette smokers compared with NS (Dougherty et al., 2013; Mitchell, 1999; Moreno et al., 2012), and also with those reporting comparable levels of trait impulsiveness between cigarette only and cannabis plus cigarette smokers (Beaton et al., 2014).

Exploratory correlations between self-reported drug use and trait impulsivity in each drug user group revealed significant positive relationships between the number of cigarettes smoked per day and total trait impulsivity in both the cigarette only and cannabis plus cigarette groups. Number of cigarettes smoked per day was also significantly positively associated with greater motor and non-planning impulsiveness in cigarette only smokers. In addition, greater nicotine dependence was significantly positively associated with trait total, motor and non-planning impulsiveness in cigarette only smokers, whilst in cannabis plus cigarette smokers only attentional impulsiveness was related to level of nicotine dependence. Therefore, whilst relationships between trait impulsivity and indicators of nicotine dependence were detected in both cigarette smoking groups, these associations differed between cigarette only and cannabis plus cigarette groups across subscales of trait impulsivity. These findings suggest that in cannabis plus cigarette smokers, greater tendencies towards distractibility and inattention may play critical roles in the transition to nicotine dependence. By contrast, in cigarette only smokers, greater tendencies towards rapid and unpremeditated behaviour may instead be integral traits that increase vulnerability for transitioning to nicotine dependence. In direct opposition, no significant positive associations were found between trait impulsivity and cannabis dependence or frequency of cannabis smoking in either cannabis group. Interestingly, motor impulsivity was found to be significantly *negatively* associated with daily frequency of cannabis smoking, providing further evidence of the limited role heightened trait impulsivity may play in cannabis use relative to cigarette smoking.

Taken together, these differences between cigarette and cannabis users in elements of trait impulsivity and in how they relate to key outcomes, suggest that a failure to assess and/or control for cigarette smoking may be responsible for inconsistencies in past research exploring trait impulsivity in cannabis smokers. Given the robust associations shown between variation in mesocorticolimbic dopamine functioning, integrity of frontostriatal circuitry and levels of trait impulsivity (e.g. Buckholtz and Meyer-Lindenberg, 2012; Volkow et al., 2011), differences in trait impulsivity observed in cigarette smokers compared with non-cigarette smokers may indicate that heightened trait impulsiveness increases the risk of tobacco cigarette smoking but may not be uniquely related to cannabis smoking. Alternatively, it is possible that trait impulsivity is influenced by prolonged cigarette smoking, suggesting trait propensities are not entirely stable constructs. It may be that regular exposure to greater levels of nicotine with or without co-morbid cannabis smoking leads to distinct changes in some dimensions of trait impulsivity as compared to cannabis smoking alone. Supporting this argument, pre-clinical studies indicate that chronic nicotine administration increases behavioural disinhibition (Kolokotroni et al., 2012) and impulsive choice (Kayir et al., 2014) in rodents. Furthermore, longitudinal assessment of other psychostimulants has found increased cocaine intake to be associated with elevated trait impulsivity on the BIS-11 while decreased intake was associated with reduced impulsivity at follow-up (Hulka et al., 2015). Against this view however, no significant positive correlations between trait impulsivity and years smoking tobacco were revealed in the present study, which would be expected if heightened trait impulsivity was a consequence of chronic nicotine-induced neural adaptations. It is imperative that future longitudinal research is conducted to determine whether trait impulsivity predates and/or is affected by extended drug use, to further understand its role in addiction and clarify its potential as an intervention target.

No significant effects of cigarette or cannabis smoking status were revealed on response inhibition. The present findings extend existing research (Filbey and Yezhuvath, 2013; Grant et al., 2012) demonstrating that capacity for response inhibition on the SST is comparable in cannabis smokers compared with non-cannabis smokers. Whilst impairments in response inhibition on the SST have been reported in cannabis smokers previously (Ramaekers et al., 2006), such observations have been limited to performance whilst under the acute influence of δ-9-tetrahydrocannibinol. Cannabis smokers in the present study on average smoked approximately 13 h before the session, suggesting that previously reported cannabis-induced impairments in response inhibition may be transient. Findings are also consistent with previous work comparing cigarette smokers and NS reporting comparable response inhibition on the SST (Bekker et al., 2005), a Stroop task (Powell et al., 2002) and an antisaccadic task (Domier et al., 2007), but not with others indicating reduced response inhibition in cigarette smokers on the go/no-go task (Luijten et al., 2011). Discrepancies in findings may be due to differences between tasks in the aspects of the behavioural inhibition they measure and the partially distinct neuropharmacological mechanisms they rely on (Eagle et al., 2008). The SST assesses action cancellation (i.e. stopping a pre-potent motoric response after it is initiated) and task performance is improved by administration of atomoxetine, a selective norepinephrine reuptake inhibitor (Broos et al., 2012). In contrast, the go/no-go task assesses action restraint (i.e. inhibiting of a pre-potent motoric response before it is initiated) and task performance is impaired following serotonin depletion (Harrison et al., 1999).

During FW trials on the IST, impaired reflection impulsivity was demonstrated in individuals who smoked cigarettes alone or cannabis alone relative to NS of either substance. Impaired reflection was demonstrated in both these drug groups compared with NS, evidenced through lower p(correct), reflective of decision-making under high levels of uncertainty, with limited information (opening fewer boxes) resulting in more incorrect judgements and fewer total points won. This heightened impulsive responding was restricted to FW trials where there were no penalties for sampling information, consistent with previous research in other drug users (Clark et al., 2006). However, there was some indication of reduced information sampling in DW trials in cigarette smokers (irrespective of cannabis use) compared with non-cigarette smokers, suggesting cigarette smoking may increase impulsive decision-making during situations involving potential risk.

Importantly, on FW trials, neither p(correct) – which is the main index of reflection impulsivity – nor number of boxes opened were altered further in those who smoked cigarettes and cannabis, suggesting co-morbid cigarette-cannabis smoking does not have additive effects on reflection impulsivity. On the contrary, those who smoked cigarettes alone made more incorrect judgements and won fewer total points than those who smoked both cigarettes and cannabis. However, the relevance of these findings is diminished given that the accuracy of judgements and total amount of points won do not directly quantify the amount of information sampled prior to decision-making (Clark et al., 2006), making these variables less valid measures of reflection impulsivity than p(correct).

Group differences were also observed in latency of box opening on the IST. During FW trials, cigarette smoking was associated with slower responses, whilst it was combined cigarette and cannabis smoking that was associated with slower responses in the DW condition. Speed of box opening has been argued to offer an insight into task-related arousal (Clark et al., 2006), suggesting that these findings may reflect differences in motivation towards the task as a function of cannabis and cigarette smoking. Reassuringly however, the group differences found in latency of box opening did not reflect those found for p(correct), suggesting any differences in motivation that may be indicated by the latency findings are unlikely to explain the differences in reflection impulsivity that were found. Furthermore, all groups sampled less information during DW than FW trials, suggesting they were sensitive to the differing reward contingencies of the task and motivated to win points.

These findings are the first to demonstrate that cigarette smoking or cannabis smoking alone significantly impair reflection impulsivity relative to no drug use. Data suggest that co-morbid cigarette-cannabis smoking does not have additive impairing effects on reflective decision-making. Findings extend previous work reporting poor reflection impulsivity in cannabis smokers where the additive effect of these drugs was not tested (Clark et al., 2009; Solowij et al., 2012). Less thoughtful decision-making may therefore be one mechanism by which cannabis smoking and cigarette smoking are independently maintained, through users being prepared to accept greater risks regarding their decisions, such as the negative health and financial consequences of their decision to smoke. Importantly, these findings have implications for drug rehabilitation. Continuing to smoke either cigarettes or cannabis whilst attempting to quit the other may sustain drug-induced impulsive behaviour leading to poorer judgements and increased likelihood of drug relapse during attempted abstinence. Indeed, previous research strongly supports this view, demonstrating cigarette smoking during cannabis abstinence significantly increases risk of relapse compared with those who do not smoke cigarettes (Haney et al., 2013). Taken together with the present study’s findings suggesting that cigarette smoking or cannabis smoking each impair reflective decision-making, it is imperative that interventions aim to stop use of both drugs in parallel to reduce risk of relapse, in line with previous suggestions (Rabin and George, 2015).

In the present study, different findings were observed between distinct dimensions of behavioural impulsivity. Differential performance on the IST and SST have been previously reported in other participant groups (e.g. Huddy et al., 2013; Jepsen et al., 2018), and is likely to reflect variations in task nature that rely on the integrity of partially distinct neurobiological systems (Caswell et al., 2015). Specifically, inhibitory control tasks involve withholding motoric responding to pre-potent stimuli, responding is highly time-sensitive, and the activity of the right inferior frontal gyrus and anterior insula is integral for inhibitory control (Aron et al., 2014; Eagle et al., 2008; Hampshire and Sharp, 2015). In contrast, the IST involves decision-making under uncertainty and responding is not time-sensitive. Although the neurobiological mechanisms underlying reflection impulsivity are currently not well defined, recent evidence suggests that inhibitory control and reflection impulsivity may be mediated by distinct physiological mechanisms (Herman et al., 2019). An important further step for future research is to explore differences between NS, cannabis only, cigarette only and cigarette plus cannabis smokers on measures of impulsive choice (e.g. delay discounting) given that this form of impulsivity is also behaviourally and neurobiologically distinct from the two dimensions studied (de Wit, 2009; Caswell et al., 2015). Behavioural impulsivity may also be greater when performance is measured in the context of drug-related rewards. Indeed, heightened response disinhibition and impulsive choice has been previously found in various drug users when drug-related stimuli are presented relative to non-drug stimuli (e.g. Pike et al., 2013; Weafer and Fillmore, 2012; Zeeb et al., 2010).

### Limitations

Despite clear distinctions in trait and behavioural impulsivity across drug user groups, some limitations of the study need to be addressed. Although cannabis groups did not differ in frequency and duration of cannabis use, cannabis plus cigarette smokers indicated significantly higher levels of cannabis dependence than cannabis only smokers on the CUDIT-R. This distinction was only present in groups being compared on trait impulsivity. A possible reason for heavier levels of cannabis dependence in cannabis plus cigarette smokers may be related to their co-morbid smoking of cigarettes, consistent with previous work indicating cigarette smoking promotes greater levels of cannabis dependence (Hindocha et al., 2015). However, the impact of this on data in the present study is likely to have been minimal given that (a) no significant correlations between trait impulsivity and cannabis dependence were found, and (b) heavier cannabis dependence reported in the cannabis plus cigarette group would bias results in favour of an additive effect of co-morbid cannabis-cigarette smoking on impulsivity, which was not found. Furthermore, previous research has indicated that level of dependence may be less accurate at predicting motivation for cannabis than weekly use (Cousijn et al., 2011), a measure that was consistent across groups in the current study.

The present findings also need to be considered in view of the fact that cannabis only smokers were not truly naïve to nicotine, as the majority of individuals in both cannabis smoking groups acknowledged using tobacco in their joints. However, this is highly consistent with European norms of cannabis smoking, where the majority of users smoke cannabis joints with tobacco (Hindocha et al., 2016; Hindocha et al., 2015). Furthermore, cannabis plus cigarette smokers reported using significantly more tobacco in their joints than cannabis only smokers, and self-reported smoking status of cannabis only smokers was further confirmed through biochemical (CO) assessment, demonstrating CO levels equivalent to a NS (Cox and Whichelow, 1985), thereby providing strong support for the legitimate classification of groups. Importantly, impulsivity differences were still observed between cannabis groups, strongly supporting the idea that despite both groups being exposed to nicotine via their joints, these individuals likely face different challenges relating to their drug use, which may in turn have implications for distinct treatment needs.

Although groups did not differ in time of last cigarette or cannabis use prior to the session, and current smoking status of individuals was supported by CO data, the amount of each drug used before the session was not restricted. Although consistent with protocols of previous investigations in these drug users (e.g. Lawn et al., 2017; Smith et al., 2011), stricter control of quantity of drug use prior to the session is an important consideration for future research. The level of alcohol use was also not controlled for in the present study. Whilst participants were excluded if they reported alcohol dependency, levels of recreational alcohol may have differed across groups and could have potentially impacted on levels of impulsive behaviour (e.g. Reed et al., 2012).

## Conclusions

Previous research attempting to understand factors underlying problematic cannabis use may have been limited by several studies overlooking the effects of co-morbid cigarette smoking. The present study was the first step towards disentangling the effects of these substances by exploring the effects of both cannabis and cigarette smoking on dimensions of trait and behavioural impulsivity in a sample of NS, cigarette only, cannabis only and cannabis plus cigarette smokers. The main findings of this study demonstrate that significantly higher trait impulsivity was found in cigarette smokers, with elevated motor impulsiveness being confined to those who did not also use cannabis. Elevated trait impulsiveness in cigarette only and cannabis plus cigarette groups may be a risk factor for the initiation of cigarette smoking and greater levels of nicotine dependence. In contrast, trait impulsivity does not appear to be related to increased cannabis smoking or dependence. Cigarette smoking or cannabis smoking appear to independently significantly impair capacity to reflect on information prior to decision-making, but interestingly, when used co-morbidly did not have an additive effect on the degree of uncertainty tolerated at the point of decision-making. In support of recent recommendations, that tobacco use should be controlled in research on health outcomes in cannabis use (Lovell et al., 2018), the present findings highlight the importance of cigarette smoking within the context of understanding the relationship between cannabis smoking and impulsivity. Treatment of cannabis dependence may benefit from tailoring interventions depending on whether individuals are co-morbid cigarette smokers; programmes targeting those who smoke both substances may benefit from ceasing use of both drugs in parallel to reduce the risk of impaired self-reflection thereby increasing likelihood of abstinence.

## References

[bibr1-0269881120926674] Adamson SJ Kay-Lambkin FJ Baker AL, et al (2010) An improved brief measure of cannabis misuse: The Cannabis Use Disorders Identification Test-Revised (CUDIT-R). Drug Alcohol Depend 110: 137–143. 20347232 10.1016/j.drugalcdep.2010.02.017

[bibr2-0269881120926674] Agrawal A Scherrer JF Lynskey MT, et al (2011) Patterns of use, sequence of onsets and correlates of tobacco and cannabis. Addict Behav 36: 1141–1147. 21820810 10.1016/j.addbeh.2011.07.005 PMC3183489

[bibr3-0269881120926674] American Psychiatric Association (2013) Diagnostic and Statistical Manual of Mental Disorders (DSM-5). Washington, D.C.: American Psychiatric Publishing, Inc.

[bibr4-0269881120926674] Aron AR Robbins TW Poldrack RA (2014) Inhibition and the right inferior frontal cortex: One decade on. Trends Cogn Sci 18: 177–185. 24440116 10.1016/j.tics.2013.12.003

[bibr5-0269881120926674] Beaton D Abdi H Filbey FM (2014) Unique aspects of impulsive traits in substance use and overeating: Specific contributions of common assessments of impulsivity. Am J Drug Alcohol Abuse 40: 463–475. 25115831 10.3109/00952990.2014.937490 PMC4318510

[bibr6-0269881120926674] Bekker EM Böcker KBE Van Hunsel F, et al (2005) Acute effects of nicotine on attention and response inhibition. Pharmacol Biochem Behav 82: 539–548. 16360813 10.1016/j.pbb.2005.10.009

[bibr7-0269881120926674] Berridge KC Robinson TE (2016) Liking, wanting, and the incentive-sensitization theory of addiction. Am Psychol 71: 670. 27977239 10.1037/amp0000059 PMC5171207

[bibr8-0269881120926674] Bonomo Y Norman A Biondo S, et al (2019) The Australian drug harms ranking study. J Psychopharmacol 33: 759–768. 31081439 10.1177/0269881119841569

[bibr9-0269881120926674] Bozkurt M Evren C Yilmaz A, et al (2013) Aggression and impulsivity in different groups of alcohol and heroin dependent inpatient men. Klin Psikofarmakol B 23: 335–344.

[bibr10-0269881120926674] Broos N Schmaal L Wiskerke J, et al (2012) The relationship between impulsive choice and impulsive action: A cross-species translational study. PloS One 7: 1–9. 10.1371/journal.pone.0036781 PMC3344935 22574225

[bibr11-0269881120926674] Buckholtz JW Meyer-Lindenberg A (2012) Psychopathology and the human connectome: Toward a transdiagnostic model of risk for mental illness. Neuron 74: 990–1004. 22726830 10.1016/j.neuron.2012.06.002

[bibr12-0269881120926674] Caswell AJ Bond R Duka T, et al (2015) Further evidence of the heterogeneous nature of impulsivity. Pers Individ Dif 76: 68–74. 25844002 10.1016/j.paid.2014.11.059 PMC4316178

[bibr13-0269881120926674] Clark L Robbins TW Ersche KD, et al (2006) Reflection impulsivity in current and former substance users. Biol Psychiatry 60: 515–522. 16448627 10.1016/j.biopsych.2005.11.007

[bibr14-0269881120926674] Clark L Roiser JP Robbins TW, et al (2009) Disrupted reflection impulsivity in cannabis users but not current or former ecstasy users. J Psychopharmacol 23: 14–22. 18515464 10.1177/0269881108089587 PMC2637477

[bibr15-0269881120926674] Congdon E Mumford JA Cohen JR, et al (2012) Measurement and reliability of response inhibition. Front Psychol 3: 1–10. 22363308 10.3389/fpsyg.2012.00037 PMC3283117

[bibr16-0269881120926674] Cousijn J Goudriaan AE Wiers RW (2011) Reaching out towards cannabis: Approach-bias in heavy cannabis users predicts changes in cannabis use. Addiction 106: 1667–1674. 21518067 10.1111/j.1360-0443.2011.03475.x PMC3178782

[bibr17-0269881120926674] Cox LS Tiffany ST Christen AG (2001) Evaluation of the brief questionnaire of smoking urges (QSU-Brief) in laboratory and clinical settings. Nicotine Tob Res 3: 7–16. 11260806 10.1080/14622200020032051

[bibr18-0269881120926674] Cox BD Whichelow MJ (1985) Carbon monoxide levels in the breath of smokers and nonsmokers: Effect of domestic heating systems. J Epidemiol Community Health 39: 75–78. 3989439 10.1136/jech.39.1.75 PMC1052406

[bibr19-0269881120926674] De Leon J Diaz FJ Becona E, et al (2003) Exploring brief measures of nicotine dependence for epidemiological surveys. Addict Behavi 28: 1481–1486. 10.1016/s0306-4603(02)00264-2 14512071

[bibr20-0269881120926674] De Wit H (2009) Impulsivity as a determinant and consequence of drug use: A review of underlying processes. Addict Biol 14: 22–31. 18855805 10.1111/j.1369-1600.2008.00129.x PMC3640851

[bibr21-0269881120926674] Dervaux A Goldberger C Gourion D, et al (2010) Impulsivity and sensation seeking in cannabis abusing patients with schizophrenia. Schizophr Res 123: 278–280. 20832995 10.1016/j.schres.2010.08.029

[bibr22-0269881120926674] Dougherty DM Mathias CW Dawes MA, et al (2013) Impulsivity, attention, memory, and decision-making among adolescent marijuana users. Psychopharmacology 226: 307–319. 23138434 10.1007/s00213-012-2908-5 PMC3581724

[bibr23-0269881120926674] Domier CP Monterosso JR Brody AL, et al (2007) Effects of cigarette smoking and abstinence on Stroop task performance. Psychopharmacology 195: 1–9. 17634928 10.1007/s00213-007-0869-x PMC2796691

[bibr24-0269881120926674] Eagle DM Bari A Robbins TW (2008) The neuropsychopharmacology of action inhibition: Cross-species translation of the stop-signal and go/no-go tasks. Psychopharmacology 199: 439–456. 18542931 10.1007/s00213-008-1127-6

[bibr25-0269881120926674] Ersche KD Roiser JP Robbins TW, et al (2008) Chronic cocaine but not chronic amphetamine use is associated with perseverative responding in humans. Psychopharmacology 197: 421–431. 18214445 10.1007/s00213-007-1051-1 PMC3785131

[bibr26-0269881120926674] Everitt BJ Belin D Economidou D, et al (2008) Neural mechanisms underlying the vulnerability to develop compulsive drug-seeking habits and addiction. Philos Trans R Soc Lond B Biol Sci 363: 3125–3135. 18640910 10.1098/rstb.2008.0089 PMC2607322

[bibr27-0269881120926674] Field M Eastwood B Bradley BP, et al (2006) Selective processing of cannabis cues in regular cannabis users. Drug Alcohol Depend 85: 75–82. 16701963 10.1016/j.drugalcdep.2006.03.018

[bibr28-0269881120926674] Filbey F Yezhuvath U (2013) Functional connectivity in inhibitory control networks and severity of cannabis use disorder. Am J Drug Alcohol Abuse 39: 382–391. 24200208 10.3109/00952990.2013.841710 PMC4318502

[bibr29-0269881120926674] Gilman JM Calderon V Curran MT, et al (2015) Young adult cannabis users report greater propensity for risk-taking only in non-monetary domains. Drug Alcohol Depend 147: 26–31. 25577478 10.1016/j.drugalcdep.2014.12.020 PMC4297698

[bibr30-0269881120926674] Goldstein RZ Volkow ND (2002) Drug addiction and its underlying neurobiological basis: neuroimaging evidence for the involvement of the frontal cortex. Am J Psychiatry 159: 1642–1652. 12359667 10.1176/appi.ajp.159.10.1642 PMC1201373

[bibr31-0269881120926674] Grant JE Chamberlain SR Schreiber L, et al (2012) Neuropsychological deficits associated with cannabis use in young adults. Drug Alcohol Depend 121: 159–162. 21920674 10.1016/j.drugalcdep.2011.08.015 PMC3242860

[bibr32-0269881120926674] Hampshire A Sharp DJ (2015) Contrasting network and modular perspectives on inhibitory control. Trends Cogn Sci 19: 445–452. 26160027 10.1016/j.tics.2015.06.006

[bibr33-0269881120926674] Harrison AA Everitt BJ Robbins TW (1999) Central serotonin depletion impairs both the acquisition and performance of a symmetrically reinforced go/no-go conditional visual discrimination. Behavi Brain Res 100: 99–112. 10.1016/s0166-4328(98)00117-x 10212057

[bibr34-0269881120926674] Haney M Bedi G Cooper ZD, et al (2013) Predictors of marijuana relapse in the human laboratory: Robust impact of tobacco cigarette smoking status. Biol Psychiatry 73: 242–248. 22939992 10.1016/j.biopsych.2012.07.028 PMC3522776

[bibr35-0269881120926674] Herman AM Critchley HD Duka T (2019) The impact of Yohimbine-induced arousal on facets of behavioural impulsivity. Psychopharmacology 236: 1783–1789. 30635680 10.1007/s00213-018-5160-9 PMC6602985

[bibr36-0269881120926674] Heatherton TF Kozlowski LT Frecker RC, et al (1991) The Fagerström test for nicotine dependence: A revision of the Fagerstrom Tolerance Questionnaire. Addiction 86: 1119–1127. 10.1111/j.1360-0443.1991.tb01879.x 1932883

[bibr37-0269881120926674] Heishman SJ Evans RJ Singleton EG, et al (2009) Reliability and validity of a short form of the Marijuana Craving Questionnaire. Drug Alcohol Depend 102: 35–40. 19217724 10.1016/j.drugalcdep.2008.12.010 PMC2694410

[bibr38-0269881120926674] Hindocha C Freeman TP Ferris JA, et al (2016) No smoke without tobacco: A global overview of cannabis and tobacco routes of administration and their association with intention to quit. Front Psychiatry 7: 1–9. 27458388 10.3389/fpsyt.2016.00104 PMC4933835

[bibr39-0269881120926674] Hindocha C Shaban NDC Freeman TP, et al (2015) Associations between cigarette smoking and cannabis dependence: A longitudinal study of young cannabis users in the United Kingdom. Drug Alcohol Depend 148: 165–171. 25622777 10.1016/j.drugalcdep.2015.01.004 PMC4337852

[bibr40-0269881120926674] Huddy VC Clark L Harrison I, et al (2013) Reflection impulsivity and response inhibition in first-episode psychosis: Relationship to cannabis use. Psychol Med 43: 2097–2107. 23339857 10.1017/S0033291712003054

[bibr41-0269881120926674] Hulka LM Vonmoos M Preller KH, et al (2015) Changes in cocaine consumption are associated with fluctuations in self-reported impulsivity and gambling decision-making. Psychol Med 45: 3097–3110. 26081043 10.1017/S0033291715001063

[bibr42-0269881120926674] Jepsen JRM Rydkjaer J Fagerlund B, et al (2018) Overlapping and disease specific trait, response, and reflection impulsivity in adolescents with first-episode schizophrenia spectrum disorders or attention-deficit/hyperactivity disorder. Psychol Med 48: 604–616. 28712363 10.1017/S0033291717001921

[bibr43-0269881120926674] Johnson MW Bickel WK Baker F, et al (2010) Delay discounting in current and former marijuana-dependent individuals. Exp Clin Psychopharmacol 18: 99–107. 20158299 10.1037/a0018333 PMC2874198

[bibr44-0269881120926674] Kagan J (1966) Reflection-impulsivity: The generality and dynamics of conceptual tempo. J Abnorm Psychol 71: 17–24. 5902550 10.1037/h0022886

[bibr45-0269881120926674] Kayir H Semenova S Markou A (2014) Baseline impulsive choice predicts the effects of nicotine and nicotine withdrawal on impulsivity in rats. Progress Neuro-Psychopharmacol Biol Psychiatry 48: 6–13. 10.1016/j.pnpbp.2013.09.007 PMC3858513 24060391

[bibr46-0269881120926674] Kolokotroni KZ Rodgers RJ Harrison AA (2012) Effects of chronic nicotine, nicotine withdrawal and subsequent nicotine challenges on behavioural inhibition in rats. Psychopharmacology 219: 453–468. 22124670 10.1007/s00213-011-2558-z

[bibr47-0269881120926674] Kolokotroni KZ Rodgers RJ Harrison AA (2014) Trait differences in response to chronic nicotine and nicotine withdrawal in rats. Psychopharmacology 231: 567–580. 24037510 10.1007/s00213-013-3270-y

[bibr48-0269881120926674] Lawn W Freeman TP East K, et al (2017) The acute effects of a dopamine D3 receptor preferring agonist on motivation for cigarettes in dependent and occasional cigarette smokers. Nicotine Tob Res 20: 800–809. 10.1093/ntr/ntx159 PMC5991206 29065193

[bibr49-0269881120926674] Logan GD Schachar RJ Tannock R (1997) Impulsivity and inhibitory control. Psychol Sci 8: 60–64.

[bibr50-0269881120926674] Lovell ME Bruno R Johnston J, et al (2018) Cognitive, physical, and mental health outcomes between long-term cannabis and tobacco users. Addict Behav 79: 178–188. 29291509 10.1016/j.addbeh.2017.12.009

[bibr51-0269881120926674] Luijten M Littel M Franken IH (2011) Deficits in inhibitory control in smokers during a go/nogo task: An investigation using event-related brain potentials. PloS One 6: 1–7. 10.1371/journal.pone.0018898 PMC3081309 21526125

[bibr52-0269881120926674] Meyer JS Quenzer LF (2013). Psychopharmacology: Drugs, the brain, and behaviour (2nd ed.). Sunderland, MA: Sinauer Associates.

[bibr53-0269881120926674] Mitchell SH (1999) Measures of impulsivity in cigarette smokers and non-smokers. Psychopharmacology 146: 455–464. 10550496 10.1007/pl00005491

[bibr54-0269881120926674] Moreno M Estevez AF Zaldivar F, et al (2012) Impulsivity differences in recreational cannabis users and binge drinkers in a university population. Drug Alcohol Depend 124: 355–362. 22425410 10.1016/j.drugalcdep.2012.02.011

[bibr55-0269881120926674] Niblett P (2018) Statistics on drug misuse England, 2018. NHS Digital Available at: https://digital.nhs.uk/data-and-information/publications/statistical/statistics-on-drug-misuse/2018 (accessed 15 November 2019).

[bibr56-0269881120926674] Patton JH Stanford MS (1995) Factor structure of the Barratt impulsiveness scale. J Clin Psychol 51: 768–774. 8778124 10.1002/1097-4679(199511)51:6<768::aid-jclp2270510607>3.0.co;2-1

[bibr57-0269881120926674] Piazza PV Deroche-Gamonet V (2013) A multistep general theory of transition to addiction. Psychopharmacology 229: 387–413. 23963530 10.1007/s00213-013-3224-4 PMC3767888

[bibr58-0269881120926674] Pike E Stoops WW Fillmore MT, et al (2013) Drug-related stimuli impair inhibitory control in cocaine abusers. Drug Alcohol Depend 133: 768–771. 24004904 10.1016/j.drugalcdep.2013.08.004 PMC3818419

[bibr59-0269881120926674] Powell J Dawkins L Davis RE (2002) Smoking, reward responsiveness, and response inhibition: Tests of an incentive motivational model. Biol Psychiatry 51: 151–163. 11822994 10.1016/s0006-3223(01)01208-2

[bibr60-0269881120926674] Rabin RA George TP (2015) A review of co-morbid tobacco and cannabis use disorders: Possible mechanisms to explain high rates of co-use. Am J Addict 24: 105–116. 25662704 10.1111/ajad.12186

[bibr61-0269881120926674] Ramaekers JG Kauert G Van Ruitenbeek P, et al (2006) High-potency marijuana impairs executive function and inhibitory motor control. Neuropsychopharmacology 31: 2296. 10.1038/sj.npp.1301068 16572123

[bibr62-0269881120926674] Reed SC Levin FR Evans SM (2012) Alcohol increases impulsivity and abuse liability in heavy drinking women. Exp Clin Psychopharmacol 20: 454–465. 23066857 10.1037/a0029087 PMC3598581

[bibr63-0269881120926674] Robbins TW Curran HV De Wit H (2012) Special issue on impulsivity and compulsivity. Psychopharmacology 219: 251–252. 22124671 10.1007/s00213-011-2584-x

[bibr64-0269881120926674] Smith AM Zunini RAL Anderson CD, et al (2011) Impact of marijuana on response inhibition: An fMRI study in young adults. J Behav Brain Sci 1: 124–133.

[bibr65-0269881120926674] Solowij N Jones KA Rozman ME, et al (2012) Reflection impulsivity in adolescent cannabis users: A comparison with alcohol-using and non-substance-using adolescents. Psychopharmacology 219: 575–586. 21938415 10.1007/s00213-011-2486-y

[bibr66-0269881120926674] United Nations Office on Drugs and Crime, UNODC (2018) World drug report 2018. Vienna, Austria: United Nations Office on Drugs and Crime.

[bibr67-0269881120926674] Van Holst RJ Schilt T (2011) Drug-related decrease in neuropsychological functions of abstinent drug users. Curr Drug Abuse Rev 4: 42–56. 21466500 10.2174/1874473711104010042

[bibr68-0269881120926674] Verbruggen F Aron AR Band GP, et al (2019) A consensus guide to capturing the ability to inhibit actions and impulsive behaviors in the stop-signal task. Elife 8: e46323. 31033438 10.7554/eLife.46323 PMC6533084

[bibr69-0269881120926674] Verbruggen F Logan GD Stevens MA (2008) STOP-IT: Windows executable software for the stop-signal paradigm. Behav Res Methods 40: 479–483. 18522058 10.3758/brm.40.2.479

[bibr70-0269881120926674] Vergés A Littlefield AK Arriaza T, et al (2019) Impulsivity facets and substance use initiation: A comparison of two models of impulsivity. Addict Behav 88: 61–66. 30145476 10.1016/j.addbeh.2018.08.018

[bibr71-0269881120926674] Volkow ND Wang GJ Fowler JS, et al (2011) Addiction: Beyond dopamine reward circuitry. Proc Natl Acad Sci 108: 15037–15042. 21402948 10.1073/pnas.1010654108 PMC3174598

[bibr72-0269881120926674] Weafer J de Wit H (2013) Inattention, impulsive action, and subjective response to d-amphetamine. Drug Alcohol Depend 133: 127–133. 23790566 10.1016/j.drugalcdep.2013.05.021 PMC3786022

[bibr73-0269881120926674] Weafer J Fillmore MT (2012) Alcohol-related stimuli reduce inhibitory control of behaviour in drinkers. Psychopharmacology 222: 489–498. 22358851 10.1007/s00213-012-2667-3 PMC4301262

[bibr74-0269881120926674] Wrege J Schmidt A Walter A, et al (2014) Effects of cannabis on impulsivity: A systematic review of neuroimaging findings. Curr Pharm Des 20: 2126–2137. 23829358 10.2174/13816128113199990428 PMC4052819

[bibr75-0269881120926674] Zeeb FD Floresco SB Winstanley CA (2010). Contributions of the orbitofrontal cortex to impulsive choice: Interactions with basal levels of impulsivity, dopamine signalling, and reward-related cues. Psychopharmacology 211: 87–98. 20428999 10.1007/s00213-010-1871-2

